# Integrated genomic, transcriptomic and metabolomic analysis reveals MDH2 mutation-induced metabolic disorder in recurrent focal segmental glomerulosclerosis

**DOI:** 10.3389/fimmu.2022.962986

**Published:** 2022-09-08

**Authors:** Qixia Shen, Lisha Teng, Yucheng Wang, Luying Guo, Feng Xu, Hongfeng Huang, Wenqing Xie, Qin Zhou, Ying Chen, Junwen Wang, Youying Mao, Jianghua Chen, Hong Jiang

**Affiliations:** ^1^ Kidney Disease Center, The First Affiliated Hospital, College of Medicine, Zhejiang University, Hangzhou, China; ^2^ Key Laboratory of Kidney Disease Prevention and Control Technology, Hangzhou, China; ^3^ Zhejiang Clinical Research Center of Kidney and Urinary System Disease, Hangzhou, China; ^4^ Institute of Nephrology, Zhejiang University, Hangzhou, China; ^5^ The Centre for Heart and Lung Innovation, The University of British Columbia, Vancouver, BC, Canada; ^6^ Department of Health Sciences Research and Center for Individualized Medicine, Mayo Clinic, Scottsdale, AZ, United States; ^7^ Dapartment of Nephrology, Shanghai Children’s Medical Center, School of Medicine, Shanghai Jiaotong University, Shanghai, China

**Keywords:** recurrent focal segmental glomerulosclerosis, kidney transplantation, whole genome sequencing, next-generation RNA sequencing, MDH2

## Abstract

Focal segmental glomerulosclerosis (FSGS) has an over 30% risk of recurrence after kidney transplantation (Ktx) and is associated with an extremely high risk of graft loss. However, mechanisms remain largely unclear. Thus, this study identifies novel genes related to the recurrence of FSGS (rFSGS). Whole genome-wide sequencing and next-generation RNA sequencing were used to identify the candidate mutant genes associated with rFSGS in peripheral blood mononuclear cells (PBMCs) from patients with biopsy-confirmed rFSGS after KTx. To confirm the functional role of the identified gene with the MDH2 c.26C >T mutation, a homozygous MDH2 c.26C >T mutation in HMy2.CIR cell line was induced by CRISPR/Cas9 and co-cultured with podocytes, mesangial cells, or HK2 cells, respectively, to detect the potential pathogenicity of the c.26C >T variant in MDH2. A total of 32 nonsynonymous single nucleotide polymorphisms (SNPs) and 610 differentially expressed genes (DEGs) related to rFSGS were identified. DEGs are mainly enriched in the immune and metabolomic-related pathways. A variant in MDH2, c.26C >T, was found in all patients with rFSGS, which was also accompanied by lower levels of mRNA expression in PBMCs from relapsed patients compared with patients with remission after KTx. Functionally, co-cultures of HMy2.CIR cells overexpressing the mutant MDH2 significantly inhibited the expression of synaptopodin, podocin, and F-actin by podocytes compared with those co-cultured with WT HMy2.CIR cells or podocytes alone. We identified that MDH2 is a novel rFSGS susceptibility gene in patients with recurrence of FSGS after KTx. Mutation of the MDH2 c.26C >T variant may contribute to progressive podocyte injury in rFSGS patients.

## Introduction

Idiopathic FSGS is the most common cause of nephrotic syndrome in which approximately 50% of patients progress to end-stage renal disease and require dialysis or kidney transplantation (KTx) within 10–20 years (1, 2). Unfortunately, in FSGS patients receiving KTx, more than 30% of cases experience recurrence of FSGS (rFSGS) with poor allograft survival and loss of re-transplantation opportunity due to the high risk of rFSGS (3–5). Several major risk factors for recurrence include younger age at diagnosis, rapid progression to end-stage renal disease, being of whiteethnicity, the loss of previous allografts due to recurrence, heavy proteinuria before the transplant, and receiving pretransplant bilateral nephrectomy (6). The most widely accepted mechanism of rFSGS after KTx involves a circulating factor. The most supportive of this theory is a rare and striking case. In that case, proteinuria rapidly developed after the kidney was transplanted into a recipient with primary FSGS. A kidney graft biopsy showed podocyte foot process effacement. Then, the doctors removed the transplanted kidney on day 14 and transplanted it into another patient with end-stage renal disease due to diabetes. Strikingly, the allografted kidney functioned well and no proteinuria was observed (7). There are many circulating factors associated with rFSGS, including soluble urokinase-type plasminogen activator receptors (8–10), cardiotrophin-like cytokine factor-1 (11), apolipoprotein A-I (12, 13), and CD40-CD40L (10, 14, 15). The release of permeability factors is potentially related to disordered circulating PBMCs (16–19). However, whether genetic variants or mutations contribute to the development of rFSGS remains unexplored.

Increasing evidence shows that integrated DNA and RNA profiling is a useful technique for genomics research to uncover the molecular mechanisms of disease and to explore the link between genotype and phenotype. RNA sequencing data can provide an orthogonal verification of DNA variant calls and can be used to prioritize expressed candidates, which are more likely to exert biological effects (20). In this study, we used high-throughput sequencing of both DNA and RNA from FSGS patients accompanied by rFSGS or remission after KTx and healthy controls (HCs) to identify genes that are potentially involved in susceptibility to rFSGS. Additionally, the functional role of the identified gene related to rFSGS was also investigated.

## Materials and methods

### Clinical sample

Three healthy kidney donors (HC group) and six adult patients with biopsies confirming primary FSGS were enrolled in this study. Of them, three recurred after KTx within one month (RC group) and three had remission for as long as 2 to 5 years (RM group). In those with rFSGS, renal follow-up biopsy was also performed. Peripheral blood from healthy individuals and patients with rFSGS or remission before and after KTx was collected from the Kidney Disease Center, The First Affiliated Hospital of Zhejiang University, China. This study was approved by the Medical Ethics Committee of The First Affiliated Hospital of Zhejiang University and the ethical batch number for it is 2021IIT065. Informed consent was obtained from all individual participants.

### Samples collection, DNA, and RNA extraction

PBMCs were isolated from 5 to 10 ml peripheral blood of KTx recipients and healthy individuals by Ficoll–Hypaque density gradient centrifugation at diagnosis of FSGS and stored at −20°C for future use. The genomic DNA was generated using the NEB Next^®^ Ultra DNA Library Prep Kit for Illumina^®^ (NEB, USA). The TRIzol Reagent (Invitrogen) and the NEBNext^®^ UltraTM RNA Library Prep Kit for Illumina^®^ (NEB, USA) were used for total RNA extraction. A NanoDrop™ 2000 (Thermo Scientific, Waltham, MA) spectrophotometer was used for a purity check, followed by agarose gel electrophoresis for degradation and contamination monitoring. A total amount of DNA of >700 ng or RNA >1 μg was at least required for library preparation and sequencing.

### Library preparation, genome, and transcriptome sequencing

NEBNext adaptor hybridization and electrophoresis were performed to select a specified length of DNA fragment. For RNA extracted, the first and second strand cDNA and NEBNext Adaptor hybridization were performed, successively. A specially designed PCR protocol was used to amplify the tagged DNA and add sequencing indexes. Then, the AMPure XP system (Beckman Coulter, Beverly, USA) was used for fragment purification. A cBot Cluster Generation System using a HiSeq 2500 PE Cluster Kit (Illumina) was used for cluster generation. Finally, the library sequencing was performed by the Illumina Hiseq 2500 platform (Illumina Inc., San Diego, CA, USA). Paired-end reads of 150 bp and 125 bp/150 bp were generated for DNA and RNA sequencing, respectively.

### SNPs analysis

We first removed the adapter in all reads and trimmed low-quality bases with a quality score below 20. Then, the clean reads were mapped to the hg19 reference genome by BWA (http://bio-bwa.sourceforge.net/). Picard was used to mark and remove duplicate reads that were generated by PCR-duplication. Per-sample BAM files were preprocessed as described in the GATK Best Practices (https://gatk.broadinstitute.org/hc/en-us). SNPs were called for every sample by the GATK variant discovery pipeline with haplotypeCaller mode. All mutations and variants were annotated according to annovar (http://annovar.openbioinformatics.org/en/latest/) and the annotation refGene in the UCSC Genome Browser. Synonymous and nonsense mutations were excluded using annotation (ANNOVAR/ONCOTATOR/SIFT).

### RNA-seq data analysis

RNA sequencing data were analyzed following the protocol reported previously (21). In brief, sequence reads were aligned to the genome using HISAT2 first. Then, Stringtie was used to assemble and quantify expressed genes and transcripts. Then, differential expression analysis was performed by the ballgown package of R software. Transcripts and genes statistically different between groups were identified by stattest using R software. TPM was used for abundance estimates.

### Enrichment analysis

Genes and transcripts with p-value <0.05, |log_2_Fold Change (FC)| >1 were selected as DEGs for further Kegg enrichment analysis by DAVID and GO enrichment analysis by Metascape, two web-based systems that incorporate information from different resources to detect the biological themes out of the candidate genes. Only pathways with Benjamini–Hochberg corrected p-value (P_BH_) <0.05 were kept as significantly enriched.

### Pathway crosstalk analysis

To describe the overlap between any given pair of pathways, two measurements were computed, the Jaccard Coefficient (JC) = (A ∩ B)/(A U B) and the Overlap Coefficient (OC) = (A ∩ B)/min(|A|, |B|), where A and B are the lists of genes included in the two tested pathways (22). To construct the pathway crosstalk, we selected a set of pathways for crosstalk analysis. Only pathways with a P_BH_ value of <0.05 were used. Meanwhile, the pathways containing fewer than three candidate genes were removed because pathways with too few genes may have insufficient biological information. Next, pathway pairs with fewer than two overlapped genes were removed. The overlap of all pathway pairs was then calculated and ranked according to their JC and OC values. The selected pathway crosstalk was visualized with the software Cytoscape.

### Western blotting

Cultured cells were lysed in RIPA Lysis Buffer (Beyotime, Shanghai, PR China) with a proteinase inhibitor cocktail, and SDS-PAGE and western blotting were performed. Results were quantified with ImageJ (National Institutes of Health, Bethesda, MD, USA). Antibodies were used to detect MDH2 (ab181873; Abcam, diluted in 1:10,000), α-tubulin (1:2,500, T9026, Sigma), synaptopodin antibody (1:1,000, ab224491, Abcam), and β-actin (1:2,000, sc-69879, Santa). The membranes were washed three times with TBST, incubated with HRP-conjugated secondary antibodies for 1 h at room temperature, visualized using a chemiluminescent substrate (Millipore, Billerica, MA), and finally analyzed using a ChemiDoc MP (Bio-Rad, Hercules, CA, USA).

### Metabolite extraction and MS analysis

Frozen cell samples in nitrogen liquid were thawed on ice, then metabolites were extracted and stored at −80°C prior to the LC–MS analysis. Pooled quality control (QC) samples were also prepared by combining 10 μl of each extraction mixture. All samples were analyzed using a TripleTOF 5600 Plus high-resolution tandem mass spectrometer (SCIEX, Warrington, UK) with both positive and negative ion modes. Chromatographic separation was performed using an ultra-performance liquid chromatography (UPLC) system (SCIEX, UK). An ACQUITY UPLC T3 column (100 mm ∗ 2.1 mm, 1.8 μm, Waters, UK) was used for the reversed-phase separation. The TripleTOF 5600 Plus system was used to detect metabolites eluted from the column. The MS data were acquired in the IDA mode. A QC sample was analyzed every 10 samples to evaluate the stability of the LC–MS.

### Metabolomics data processing

The acquired LC–MS data pretreatment was performed using XCMS software. Raw data files were converted into mzXML format and then processed using the XCMS, CAMERA, and metaX toolbox included in the R software. Each ion was identified by the comprehensive information of retention time and m/z. The intensity of each peak was preprocessed using metaX. The open-access databases, KEGG and HMDB, were used to annotate the metabolites. Features that were detected in <50% of QC samples or 80% of test samples were removed, and values for missing peaks were extrapolated with the k‐nearest neighbor algorithm. Then, PCA, data normalization, and QC-robust spline batch correction were performed. The P-value was analyzed by Student t‐tests, which were adjusted for multiple tests using an FDR (Benjamini–Hochberg). We also conducted the supervised PLS‐DA using the metaX to variable discriminant profiling statistical method to identify more specific differences between the groups. The VIP cut‐off value of 1.0 was set to select important features.

### Cell culture and treatment

Because B cells play an important role in rFSGS (4, 23–27) and HMy2.CIR is a human B lymphoblastoid cell line, to confirm the functional role of the identified gene with the MDH2 c.26C >T mutation, a homozygous MDH2 c.26C >T mutation in HMy2.CIR cell line was induced by CRISPR/Cas9 and co-cultured with podocytes, mesangial cells, or HK2 cells, respectively. Briefly, WT and MDH2 c.26C >T mutant Human HMy2.CIR cells were bought from Cyagen (Guangzhou, China). The HMy2.CIR cells were cultured in IMDM (12440053, Gibco), HK2 cells in DMEM/F12(D8437, Sigma), human mesangial cells (HMC) in DMEM/HG (D6429, Sigma) containing 10% FBS (12103C, Sigma) and 1% penicillin–streptomycin (15070063, Gibco), incubated at 37°C in a humidified atmosphere containing 5% CO2, and routinely passaged every 2 or 3 days. Podocytes were first grown under proliferation-permissive conditions in the presence of 33°C in a humidified atmosphere of 5% CO2. Growth media consists of RPMI 1640 (R8758, Sigma) containing 10% FBS and 1% penicillin-streptomycin supplemented with 100× Insulin-Trans-Sel-G (41400045, Gibco). For differentiation, they were switched to type I collagen-coated (354236, Corning) culture dishes deprived of ITS-G and half FBS for 7–9 days at 37°C. For Western blot, 3 × 10^4^ podocytes, HMC, or HK2 cells were seeded in 6-well plates until 70% converged and then co-cultured with 5 × 10^4^ WT HMy2.CIR or mutant HMy2.CIR cells for 48 h. For immunofluorescence, 10^4^ podocytes or HMC were seeded in 24-well plates until 70% converged and then co-cultured with 10^4^ WT HMy2.CIR or mutant HMy2.CIR cells for 48 h.

### Cell cycle arrest

Approximately 2 × 10^5^ serum-starved WT HMy2.CIR or mutant HMy2.CIR cells were washed with phosphate buffer saline before being fixed with 70% cold ethanol and kept at 4°C for 3.5 h. Phosphate buffer saline was used to wash cells again and resuspend cells. Cells were subjected to propidium iodide staining with a Cell Cycle and Apoptosis Analysis Kit (C1052, Beyotime), and the DNA content was measured by a fluorescence-activated cell sorting instrument (BD FACSCanto II) and analyzed by Modfit LT5.0.

### Real-time quantitative PCR

Total RNA was extracted from HMC and HK2 cells by Trizol reagent (Invitrogen, USA). cDNA was prepared using the PrimeScriptTM RT reagent Kit with gDNA Eraser (No. RR047A, TaKaRa, Japan) as per the protocol of the manufacturer. Real-Time PCR was run using SYBR Green and CFX96™ Real-Time PCR Detection Systems (Bio-Rad, CA, USA) using the primers in **Table S1**. The mRNA levels of selected genes were calculated after normalization to GAPDH by using the 2^−ΔΔCt^ method as per the protocol of the manufacturer.

### Immunofluorescence assessment of cultured podocytes

Coverslips containing podocytes were fixed with 4% paraformaldehyde for 15 min and blocked in PBS containing 0.1% Triton X-100 and 3% bovine serum albumin(BSA) for 30 min at room temperature prior to incubation with synaptopodin antibody (1:100, sc-21537, Santa), NPHS2 antibody (1:100, ab50339, Abcam), Megsin (1:100, bs-0815R, Bioss) overnight in a humidified chamber at 4°C. Slides were incubated with secondary antibody Alexa Fluor 594-conjugated anti-goat IgG (1:500), Alexa Fluor 594-conjugated anti-rabbit IgG (1:500), and FITC-phalloidin (1:100) (P5282, Sigma) for 1 h at 37°C to stain the cytoskeleton. Sections were then examined by immunofluorescence microscopy (Leica DMLB, Wetzlar, Germany).

### Statistical analysis

Results are displayed as means ± SEM. Statistical analysis using Student’s t‐tests was performed with GraphPad Prism 7 (GraphPad Inc, San Diego, CA, USA). p  < 0.05 was considered statistically significant.

## Results

### Clinical and pathological characteristics of rFSGS patients

**Figure 1:**
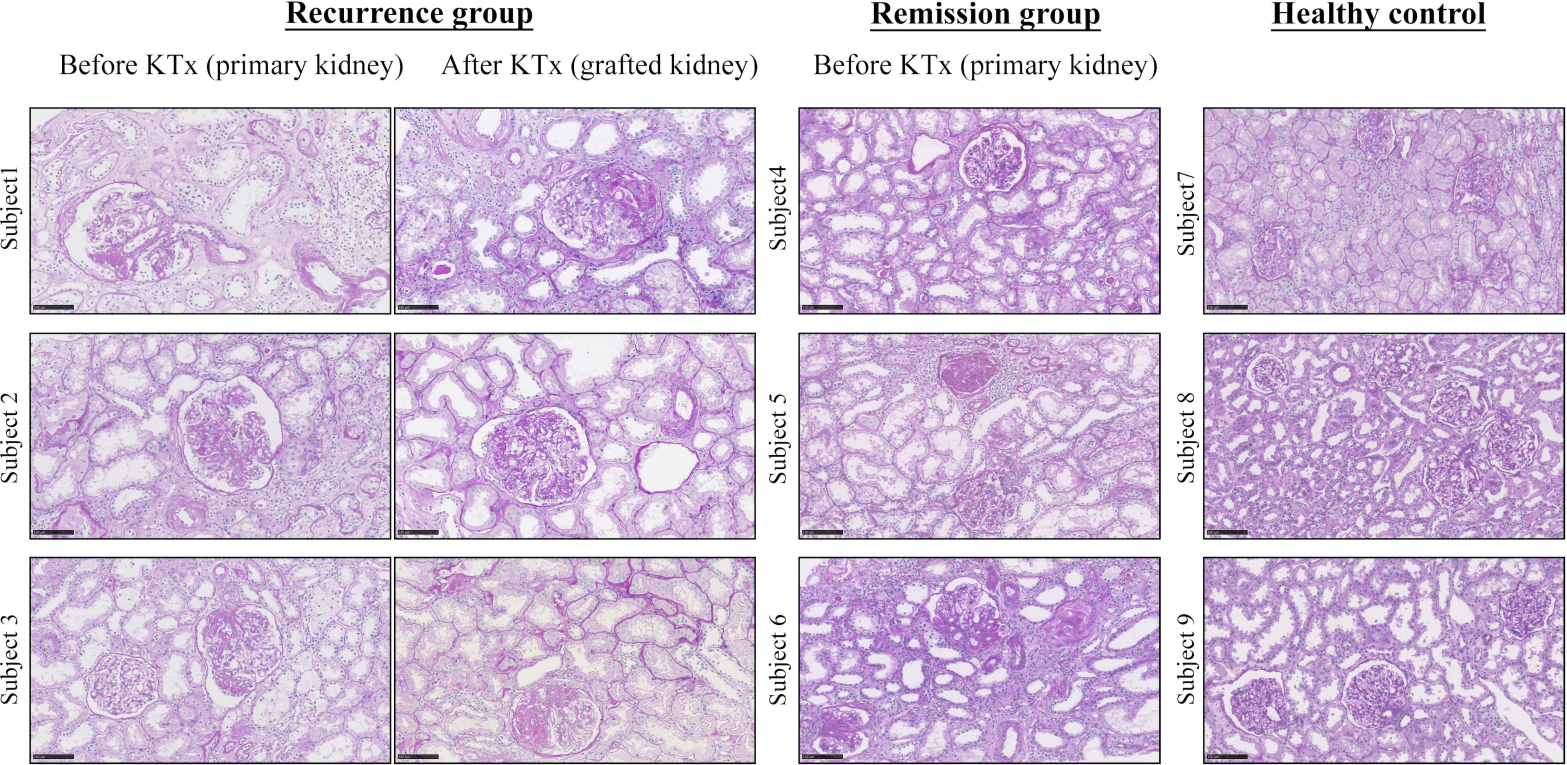
Representative pathology from FSGS patients before and after KTx by Periodic acid-Schiff staining. Note that focal segmental glomerular sclerosis with fibrosis is apparent in all FSGS patients before KTX and is also found in those with recurrent FSGS patients after KTx. Because KTx largely improved progressive renal injury in those FSGS patients, no renal biopsy was performed in the remission patients after KTx based on the ethical reasons. The healthy control kidneys are obtained from unmatched donor kidneys. Scale bar: 100um. KTx, kidney transplantation.

A total of six FSGS patients after KTx were enrolled in this study, including three with rFSGS and three with remission. In addition, three healthy kidney donors were also included as a normal control. Graft biopsy was performed in all patients. Clinically, proteinuria recurred in all patients with rFSGS but not in those with remission, which is shown in **Table S2**. All patients with FSGS showed severe FSGS with extensive tubulointerestial fibrosis as shown in renal biopsied kidneys before KTx (**Figure 1**). In patients with rFSGS, KTx did not improve renal dysfunction, with similar FSGS pathology found in the grafted kidneys as before KTx (**Table S2**; **Figure 1**). In contrast, in patients with FSGS remission, progressive renal injury was largely improved after KTx. During follow-up, these patients did not develop significant edema and proteinuria. All patients declared non-familial FSGS history.

### Identification of candidate SNPs and genes associated with rFSGS

**Figure 2:**
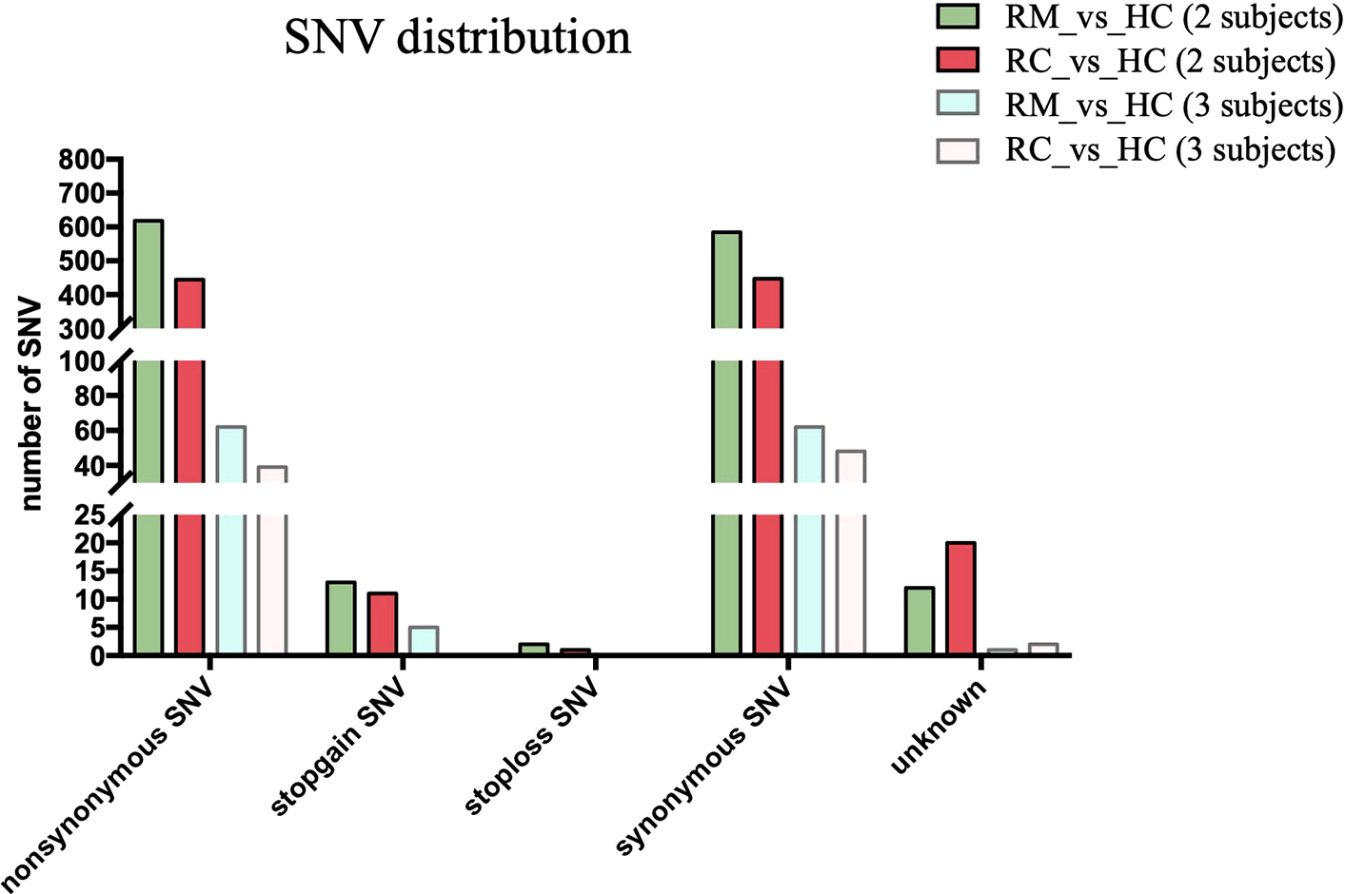
Identification of SNPs related to recurrence of FSGS after kidney transplantation. Bar plot showing summary of different types of SNP variants mapped to exons identified in PBMCs from recurrence (n≥2) and remission (n≥2) subjects excluding those also identified in HCs. RC, recurrence; RM, remission; HC, healthy control.

**Figure 3:**
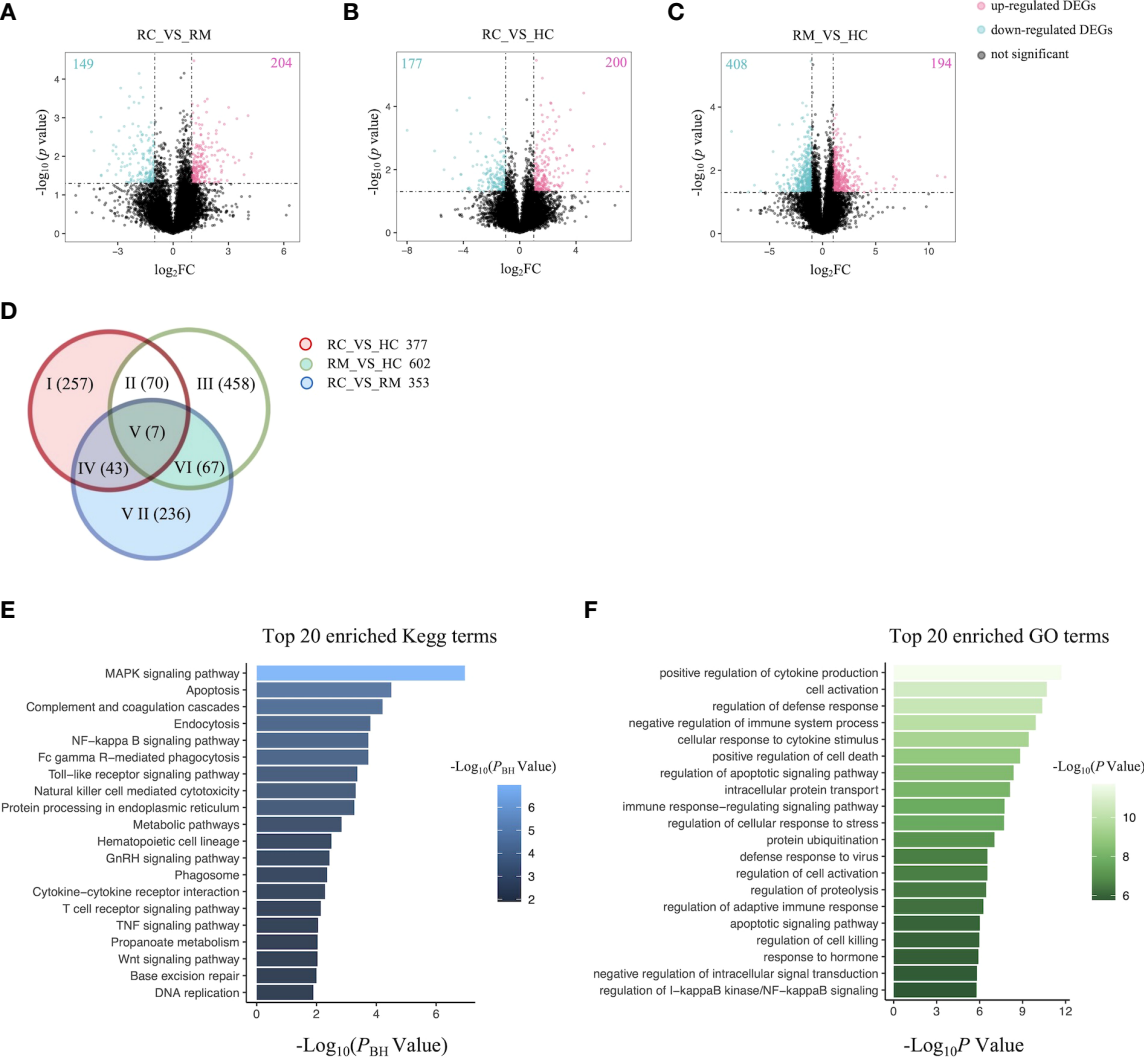
Identification of DEGs related to recurrence of FSGS after kidney transplantation.Volcano plot showing DEGs between **(A)** RC group and RM group; **(B)** RC group and HC group; **(C)** RM group and HC group. Each dot represents a gene. Red dots represent up-regulate genes, green dots represent down regulated genes. The numbers marked in the upper left and upper right corner represents the number of DEGs. **(D)** Venn diagram showing overlap of DEGs identified by pairwise comparison among the three groups. Top 20 enriched **(E)** Kegg terms and **(F)** GO terms. RC, recurrence; RM, remission; HC, healthy control.

We next performed both whole-genome sequencing and transcriptomic analysis to identify the potential genetic variants responsible for rFSGS. Genome sequences of hg19 were treated as references. **Figure 2** showed the number and type of SNPs presented in exons from ≥2 recurrent or remission subjects but absent in any healthy controls. We therefore identified 1,012 and 1,360 somatic SNPs in recurrent or remission subjects, respectively. Of them, 39 and 62 were nonsynonymous SNPs presented in three recurrent or remission patients, respectively. Only nonsynonymous SNP variants presented in all three recurrence patients but in ≤1 remission patient and none of the HCs were regarded as associated with rFSGS after KTx. Thus, a total of 32 nonsynonymous SNP variants mapped to exons across 29 unique genes were selected (**Table S3**). The pairwise comparison was conducted for the transcriptional patterns of PBMCs from three groups (**Figures 3A–C**). Approximately 353 DEGs between the recurrence and remission groups (**Figure 3D**), as well as 300 DEGs between the recurrence group and HCs, excluding those also differentially expressed between remission subjects and HCs (**Figures 3D**), were deemed as genes related to rFSGS after KT.

### The immune and metabolic pathways are significantly altered in patients with rFSGS

**Figure 4:**
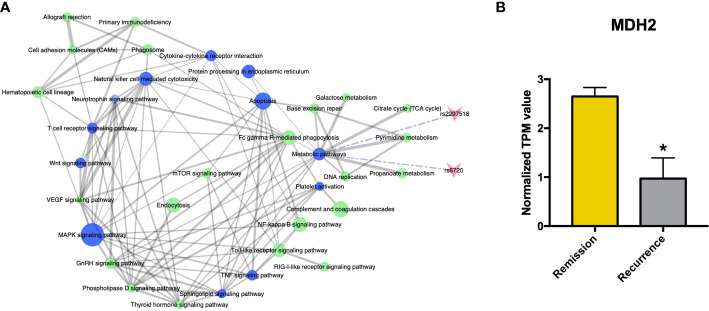
Crosstalk analysis among significantly enriched pathways suggested homozygous c.26C>T variant in MDH2 as a potential pathogenic mutation. **(A)** Pathway crosstalk among rFSGS related pathways. Triangular arrow represent SNPs, blue nodes represents pathways involving SNPs, green nodes represent pathways without SNPs involvement. Solid line represents crosstalk between each pair, Edge-width corresponds to the score of specific pathway pair. Larger edge-width indicates higher score. Dotted line represents SNPs enrichment. **(B)** mRNA expression level of MDH2 in remission group and recurrence group. *p <0.05.

The functional features of the rFSGS-related genes were examined by Kegg pathway enrichment on DAVID and GO terms enrichment on Metascape. A total of 65 significantly kegg pathways were enriched by DAVID. The top 20 enriched Kegg pathways and GO terms are shown in **Figures 3E, F**, respectively. Of 65 enriched Kegg pathways, 34 with three or more DEGs overlapping with one another were extracted (**Table S4**). All the pairs formed by these pathways were utilized to build the pathway crosstalk and were analyzed according to the average scores of coefficients JC and OC. Results shown in **Figure 4A** revealed the immune-related crosstalk pathways, including MAPK signaling pathways, complement, and coagulation cascades, NF-kappa B signaling pathway, Toll-like receptor signaling pathway, TNF signaling pathway, Wnt signaling pathway, etc., and the metabolism-related crosstalk pathways, such as citrate cycle, pyrimidine metabolism, galactose metabolism, etc. Of note, these immune and metabolism-related pathways were also widely connected to form the signaling pathways network. Among the top 20 enriched GO terms, positive regulation of cytokine production, cell activation, negative regulation of immune system processes, and cellular response to cytokine stimulus are ranked at the top. This is supportive of our hypothesis that circulating factors released by disturbed PBMCs may be causative of rFSGS.

### rs6720 variant in MDH2 was identified as a candidate mutation associated with rFSGS

There were two rFSGS related SNPs-mapped genes enriched in the crosstalk pathways (**Table S3**; **Figure 4A**), including rs6720 (mapped to MDH2, C >T, homozygote) and rs2297518 (mapped to NOS2, G >A, heterozygote). Both SNPs uniquely existed in all three recurrent recipients but in none of the remisson recipients or healthy donors. Furthermore, MDH2 was confirmed to be a DEG between the recurrence group and the remission group (**Figure 4B**), whereas NOS2 was not. MDH2 is a gene that encodes the mitochondrial malate dehydrogenase enzyme involved in the Krebs cycle. Thus, the rs6720 variant in MDH2 may be associated with the disorder in metabolic pathways and the Krebs cycle (**Table S3**).

### rs6720 variant in MDH2 caused loss of protein expression in HMy2.CIR cells

**Figure 5:**
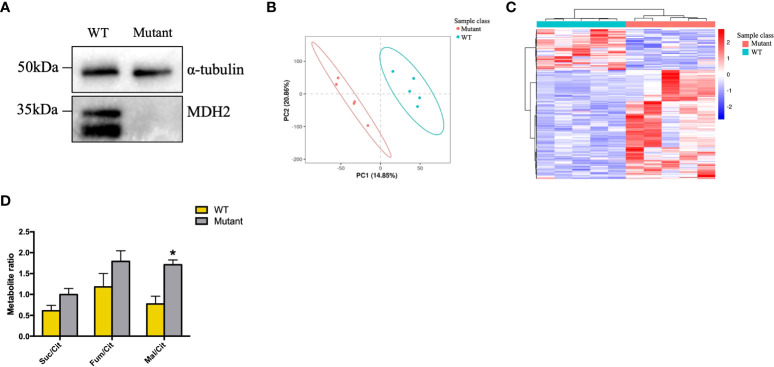
rs6720 leads to loss of MDH2 expression in HMy2.CIR cells and disturbed crebs cycle. **(A)** Western blot analysis with anti-MDH2 antibodies in rs6720 mutant and WT HMy2.CIR cells. a-tubulin was used as loading controls. **(B)** The scores plot of PLS-DA model of the LS-MS (positive model) spectral data between the selected PCs. Each point in the graph represents a sample. Different colors represent different groups, and different color circles represent different PCA groups. **(C)** Results of clustering patterns of metabolites differential expression. Red represents upregulation, blue represents downregulation. Different color bars represent different groups. **(D)** Metabolite ratios assessed by liquid chromatographic tandem-mass spectrometry in mutant and WT HMу2.CIR cells. Suc, succinate; Cit, citrate; Mal, malate; *p<0.05.

Human PBMCs exist in a heterogeneous pool comprised of 70% T cells, 10% B cells, and another 10% of monocytes. Analysis of the kidney biopsy samples from children with idiopathic nephrotic syndrome revealed a significantly higher number of glomerular CD20^+ ^B cell infiltration in FSGS patients (23). B-cell depleting antibodies like Rituximab and Ofatumumab are effective in rFSGS (4, 24–27), which indicates that the recurrence of FSGS may be B-cell related. HMy2.CIR, a human B lymphoblastoid cell line, was thus chosen to detect the effect of the homozygous c.26C >T variant on MDH2 protein expression. Indeed, substantially lower levels of MDH2 protein were detected in HMy2.CIR cells carrying the c.26C >T mutation than in WT controls (**Figure 5A**). Moreover, we also found the proliferation rate of mutant HMy2.CIR cells were significantly lower than that of WT cells, as the percentage of S phase was significantly reduced in mutant HMy2.CIR cells compared to WT cells (**Figures S1A, B**). Next, we performed a metabolomic analysis to examine whether the mutation of MDH2 in HMy2.CIR cells may lead to metabolic alterations. PLS-DA was applied in the data analysis and the score plot showed that the metabolomic profile of mutant cells was significantly altered when compared to the WT cells (**Figures 5B, C**). Furthermore, metabolic changes could be observed by Kegg enrichment of the differential metabolites (**Figure S2**). As shown in **Figure 5D**, the ratio of malate to citrate was significantly increased in mutant cells than in WT control, and the ratio of fumarate, a direct precursor of malate, to citrate was also higher in mutant cells than in WT control, although no statistical significance was detected. In contrast, mutation of MDH2 resulted in a decrease in the ratio between succinate and citrate without statistical significance (**Figure 5D**).

### MDH2 variants in HMy2.CIR cells caused injury to podocytes specifically

**Figure 6:**
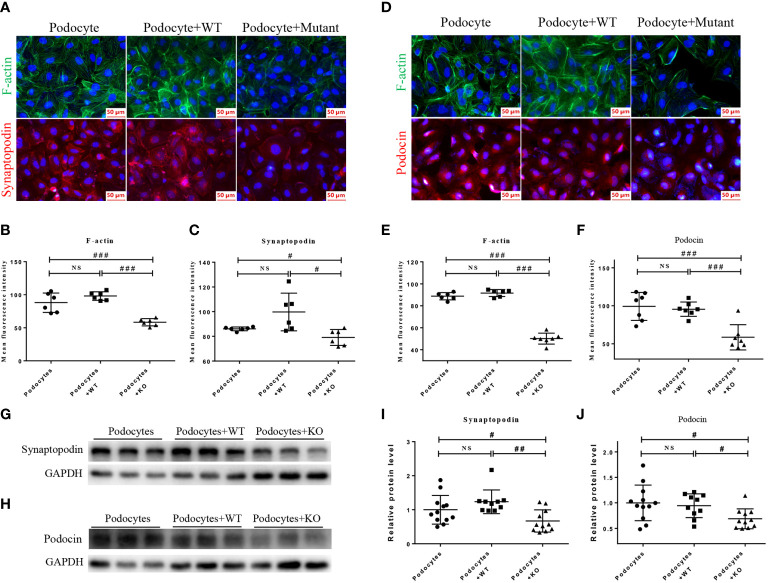
HMy2.CIR cells carrying rs6720 mutation in MDH2 caused injury to podocytes. **(A–C)** Representative pictures and quantification of immunofluorescence staining of F-actin (green), synaptopodin (red) and DAPI (blue) in podocytes before and after WT or mutant HMy2.CIR cell exposure. **(D–F)** Representative pictures and quantification of immunofluorescence staining of F-actin (green) and podocin (red) in podocytes before and after WT or mutant HMy2.CIR cell exposure. Six random fields were taken from each coverslip (mean ± SD, n=6). Scale bar: 50um. Western blot and statistics for Synaptopodin **(G, I)** and Podocin **(H, J)** from podocytes before and after WT or mutant HMy2.CIR cell exposure (mean ± SD, n=9-12). ^#^p < 0.05; ^##^p<0.01; ^###^p<0.001; NS, no significance.

Because podocyte injury is a recognized cause of FSGS (28), we next examined the functional role of mutant MDH2 in podocyte injury by co-culturing mutant or WT HMy2.CIR cells with human podocytes, HMC, and HK2 cells, respectively. Immunofluorescence and western blot analysis showed that cells cultured with mutant HMy2.CIR resulted in podocyte injury as demonstrated by reducing podocyte-specific markers including expression of F-actin, synaptopodin, and podocin (**Figure 6**). This is in accordance with the biopsy results under the electron microscope. Podocyte foot process effacement was observed in all rFSGS patients, ranging from partially to diffusely (**Table S2**). In contrast, no significant changes were found in the co-culture of mutant HMy2.CIR with HMC (**Figures S3A, B**) and HK2 cells (**Figure S4**), which showed no alteration in expression of megsin (a HMC marker) that is involved in the renal tissues of various glomerular diseases (29) and expression of IL1β, RANTES, MCP-1, and TIM-1 by HK2 cells. Taken together, findings from these studies implied the restricted pathogenicity of c.26C> T variants in MDH2 to podocytes.

## Discussion

FSGS is a pathologic rather than a diagnostic CKD, which can be caused by rare, highly penetrant mutations in a number of genes (30). Injury to the podocytes plays a central role in the pathogenesis of FSGS (31, 32). Increasing evidence shows that mutations in genes related to FSGS are found in podocytes, including those involved in slit diaphragm structure and function, actin cytoskeleton, or cell signaling apparatus (33, 34). By integrated genomic and transcriptomic analysis, we reported here that rFSGS patients carried a homozygous c.26C> T mutation in the MDH2 gene in PBMCs with metabolic and immune dysfunction. However, the pathogenic role of mutant MDH2 in the PBMCs of rFSGS patients remains largely unknown. MDH2 encodes the enzyme malate dehydrogenase, mitochondrial in humans, which catalyzes the reversible oxidation of malate to oxaloacetate by utilizing the NAD-NADH cofactor system in the Krebs cycle. Despite the assumption that loss-of-function mutations affecting this key enzyme appear to be typically incompatible with life, pathogenic MDH2 disorder has been reported in several studies. A loss-of-function germline mutation in MDH2 was observed in paraganglioma (35). Bi-allelic mutations in MDH2 were also reported to cause the early-onset of severe encephalopathy (36). A prognostic model based on nine signature glycolytic genes, including MDH2, can accurately predict the prognosis of brain glioma patients. Among them, the mutation rate of HDAC4 and MDH2 genes was the highest at 8% (37). MDH2 was also overexpressed in endometrial carcinoma tissues and was related to the grade of the cancer (38). In renal cell carcinoma, inhibition of the glutamine-MDH2 axis suppresses *in vitro* tumor phenotypes in an L-2-HG-dependent manner (39). Decreased protein levels of malate dehydrogenase 2 were detected in a mouse model of methylmalonic aciduria, an inborn metabolic disorder of propionate catabolism that may lead to metabolic stroke and renal insufficiency (40). It was believed that disruption of enzymatic activities like malate dehydrogenase 2 could lead to the accumulation of specific metabolites, which give rise to epigenetic changes in the genome that cause a characteristic hypermethylated phenotype (41).

Functionally, we also uncovered that the mutation in the MDH2 gene was able to specifically cause podocyte injury. This was demonstrated by the finding that co-culture of human HMy2.CIR cells with rs6720 point mutation in MDH2 with podocytes were able to cause podocyte injury by inhibiting the expression of F-actin, synaptopodin, and podocin. Whereas no injuries were evidenced in by co-culturing the HMy2.CIR cells with other kidney cell types including human mesangial cells (HMC) and tubular epithelial cells (HK2). Thus, results from this study uncovered the restricted pathogenicity of MDH2 c.26C> T variants to podocyte injury specifically.

It should be pointed out that there are several limitations to this study. First, although integrated genomic and transcriptomic analysis was performed, this study is limited by the number of patients enrolled. Further studies on rs6720 in MDH2 leading to rFSGS in a large-scale cohort are necessary to confirm our findings. Second, though MDH2 abrogation leads to increased levels of malate/citrate ratios, as was proved in HMy2.CIR cells, we have not observed the absence of malate accumulation in rFSGS patients. Furthermore, the mechanisms underlying the pathogenicity of HMy2.CIR cells carrying homozygous c.26C> T mutations in podocyte injury has not been clarified. We speculated that the abnormal protein expression caused by the c.26C> T mutation in MDH2 led to the disorder of the TCA cycle and disturbance of cell metabolism, then the production of abnormal circulating factors by immune cells. These abnormal circulating factors resulted in podocyte injury, which is presented as inhibition of the expression of F-actin, synaptopodin, and podocin. Our next step is to include more rFSGS cases and screen out the possible circulation factors. The mechanism underlying podocyte injury should then be further investigated.

In summary, by using integrated genomic and transcriptomic analysis, we identified for the first time a bi-allelic c.26C >T variant in MDH2 in patients with rFSGS. Furthermore, we also found that this mutation is pathogenic and can cause podocyte injury specifically.

## Data availability statement

The data presented in the study are deposited in the SRA repository, accession number PRJNA842944, and the GEO repository, GSE212610.

## Ethics statement

The studies involving human participants were reviewed and approved by the Medical Ethics Committee of The First Affiliated Hospital of Zhejiang University. The patients/participants provided their written informed consent to participate in this study.

## Author contributions

QS and HJ conceived and designed the experiments and drafted the manuscript. QS, HH, WX, YC, JC, and QZ collected human samples. QS, LT, LG, and FX performed the experiments and data analysis. QS, LT, and HJ interpreted the results of the experiments. QS, JW, YM, and HJ edited and revised the manuscript. QS and LT prepared the figures. HJ and QS approved the final version of the manuscript. All authors listed have made a substantial, direct, and intellectual contribution to the work and approved it for publication.

## Funding

This work was partially supported by grants from NSFC U21A20350, 82070767, 81970651, and 82070729.

## Conflict of interest

The authors declare that the research was conducted in the absence of any commercial or financial relationships that could be construed as a potential conflict of interest.

## Publisher’s note

All claims expressed in this article are solely those of the authors and do not necessarily represent those of their affiliated organizations, or those of the publisher, the editors and the reviewers. Any product that may be evaluated in this article, or claim that may be made by its manufacturer, is not guaranteed or endorsed by the publisher.

## Supplementary material

Supplementary Table 1 list of primer sequences used in this study.

Supplementary Table 2 Characteristics of included subjects.

Supplementary Table 3 list of non-synonymous SNPs related to rFSGS.

Supplementary Table 4 Pathways included for crosstalk analysis.
