# Supplementary material for: Integrated genomic, transcriptomic and metabolomic analysis reveals MDH2 mutation-induced metabolic disorder in recurrent focal segmental glomerulosclerosis
Source: Front Immunol. 2022 Sep 8;13:962986. doi: 10.3389/fimmu.2022.962986 (PMC9495259; doi:10.3389/fimmu.2022.962986)
Supplement: Supplementary Table 1 — list of primer sequences used in this study. [file Table_1.docx]

| **Table S1** list of primer sequences used in this study | | |
| --- | --- | --- |
| **Gene** | **Primer Forward (5`-3`)** | **Reverse (5`-3`)** |
| COL1A1 | TGGTGCTACTGGTGCTGC | CACCCTGGGGACCTTCAGAG |
| MCP-1 | GACCATTGTGGCCAAGGAGA | TTGGGTTTGCTTGTCCAGGT |
| IL1β | CAGAAGTACCTGAGCTCGCC | AGATTCGTAGCTGGATGCCG |
| RANTES | CAGTCGTCTTTGTCACCCGA | TCTTCTCTGGGTTGGCACAC |
| TIM-1 | AGGAGTCAGTTTGGCGGTTA | GAGAGCTCTGTGCCTTCCAA |
| GAPDH | CCATGGGGAAGGTGAAGGTC | GCGCCCAATACGACCAAATC |
