# Supplementary material for: Integrated genomic, transcriptomic and metabolomic analysis reveals MDH2 mutation-induced metabolic disorder in recurrent focal segmental glomerulosclerosis
Source: Front Immunol. 2022 Sep 8;13:962986. doi: 10.3389/fimmu.2022.962986 (PMC9495259; doi:10.3389/fimmu.2022.962986)
Supplement: Supplementary Table 2 — Characteristics of included subjects. [file Table_2.docx]

| **Table S2** Characteristics of included subjects | | | | | | | | | |
| --- | --- | --- | --- | --- | --- | --- | --- | --- | --- |
|  | **Recurrence group** | | | **Remission group** | | | **Healthy control group** | | |
|  | Subject 1 | Subject 2 | Subject 3 | Subject 4 | Subject 5 | Subject 6 | Subject 7 | Subject 8 | Subject 9 |
| Gender | Male | Male | Male | Male | Male | Female | Female | Female | Female |
| Age | 42 | 25 | 26 | 61 | 54 | 49 | 56 | 66 | 68 |
| Weight | 78 | 62 | 52 | 70 | 73 | 52 | 46 | 53 | 51 |
| Duration of HD  before KTx | 6 months | 1 year | 1year | 11 months | 13 months | 2 years | NA | NA | NA |
| eGFR  (^*^D/R, mL/min) | NA /23.39 | NA / 25.13 | NA / 12.89 | 10.3/NA | 3.3/NA | 9.6 / NA | 99.17/NA | 93.97/NA | 82.86/NA |
| Proteinuria  (^*^ D/R, g/day) | NA / 1.53 | NA / 3.43 | NA / 2.78 | 7.54 / NA | 5.81 / NA | 3.27 / NA | 0.05 / NA | 0.07 / NA | 0.08 / NA |
| Creatinine  (^*^ D/R, µmol/L) | 134 / 361 | 172 / 348 | 113 / 298 | 139 / NA | 136 / NA | 159 / NA | 62 / NA | 59 / NA | 67 / NA |
| Total cholesterol  (mmol/L) | 2.07 | 2.05 | 2.67 | 1.86 | 1.55 | 4.01 | 4.55 | 5.37 | 5.23 |
| Hemoglobin (g/L) | 90 | 89 | 78 | 104 | 90 | 94 | 135 | 134 | 133 |
| Urea Nitrogen (mg/dL) | 36.0 | 32.5 | 29.5 | 11.53 | 2.38 | 9.54 | 5.0 | 6.0 | 4.0 |
| Total calcium (mmol/L) | 1.93 | 1.84 | 2.16 | 1.89 | 2.38 | 2.34 | 2.42 | 2.28 | 2.17 |
| Albumin  (g/L) | 43.6 | 47.6 | 53.7 | 48.7 | 51.6 | 52.4 | 37.6 | 48.9 | 47.9 |
| No. of glomeruli (total/sclerotic) | 37 / 17 | 41 / 25 | 39 / 17 | 28 / 3 | 38 / 7 | 33 / 5 | 21 / 0 | 25 / 0 | 30 / 0 |
| Characteristics of kidney grafts under electron microscope | Foot process diffusely fused, basal membrane segments thickened, mesangial matrix increased, and electron dense sediments were not found | Most of the foot processes are fused, the basement membrane is normal, the mesangial matrix increases, and no deposition is found | The foot process is partially fused, the basement membrane is acceptable, the mesangial matrix increases, no compactness is found | NA | NA | NA | NA | NA | NA |
| Family history of kidney diseases | No | No | No | No | No | No | No | No | No |
| Donor type | Deceased | Living | Living | Living | Living | Deceased | NA | NA | NA |
| immunosuppressive regimen after KTx | FK506+MMF  +Prednisone | FK506+MMF  +Prednisone  +Thymoglobuline | FK506+MMF  +Prednisone | FK506+MMF  +Prednisone | FK506+MMF  +Prednisone | FK506+MMF  +Prednisone | NA | NA | NA |

KTx, kidney transplantation; ^*^ D/R: at diagnosis/at recurrence; MMF, mycophenolate mofetil; NA, not applicable.
