# Supplementary material for: Integrated genomic, transcriptomic and metabolomic analysis reveals MDH2 mutation-induced metabolic disorder in recurrent focal segmental glomerulosclerosis
Source: Front Immunol. 2022 Sep 8;13:962986. doi: 10.3389/fimmu.2022.962986 (PMC9495259; doi:10.3389/fimmu.2022.962986)
Supplement: Supplementary Table 3 — list of non-synonymous SNPs related to rFSGS. [file Table_3.docx]

| **Table S3** list of non-synonymous SNPs related to rFSGS | | | | |
| --- | --- | --- | --- | --- |
| **GENE** | **SNP ID** | **former** | **mutation** | **type** |
| ADAM28 | rs7829944 | G | C | heterozygotes |
| ARHGAP30 | rs3813609 | G | C | heterozygotes |
| C7orf57 | rs10233232 | T | C | heterozygotes |
| C7orf57 | rs2708890 | C | A | heterozygotes |
| C7orf57 | rs2708912 | C | T | heterozygotes |
| EXOC3L4 | rs744153 | C | G | homozygotes |
| FAM124B | rs3738954 | A | G | heterozygotes |
| FCGBP | rs201020361 | G | A | heterozygotes |
| GGCX | rs699664 | C | T | homozygotes |
| HSD3B1 | rs1047303 | C | A | heterozygotes |
| ISM2 | rs3742728 | C | T | heterozygotes |
| LURAP1L | rs3750501 | A | G | heterozygotes |
| MAN2B2 | rs2301788 | A | G | homozygotes |
| MAN2B2 | rs2301796 | A | C | homozygotes |
| MAN2B2 | rs2301790 | A | G | heterozygotes |
| MDH2 | rs6720 | C | T | heterozygotes |
| METTL22 | rs2270286 | G | C | heterozygotes |
| NBPF1 | rs200783506 | G | A | heterozygotes |
| NOS2 | rs2297518 | G | A | heterozygotes |
| PTCH1 | rs357564 | G | A | homozygotes |
| RNF181 | rs6643 | T | C | homozygotes |
| SLC9A3 | rs2247114 | A | G | homozygotes |
| SYDE2 | rs56191061 | G | A | heterozygotes |
| TMEM241 | rs8099409 | G | A | heterozygotes |
| UMODL1 | rs3819142 | A | C | heterozygotes |
| WDR87 | rs73027451 | C | T | heterozygotes |
| WHAMM | rs3814281 | G | A | heterozygotes |
| ZFHX3 | rs4788682 | A | G | homozygotes |
| ZNF607 | rs12461753 | G | A | heterozygotes |
| ZNF607 | rs386809063 | G | A | heterozygotes |
| ZNF83 | rs1056185 | C | T | homozygotes |
| ZNF875 | rs3745764 | C | T | heterozygotes |
