# Supplementary material for: Integrated genomic, transcriptomic and metabolomic analysis reveals MDH2 mutation-induced metabolic disorder in recurrent focal segmental glomerulosclerosis
Source: Front Immunol. 2022 Sep 8;13:962986. doi: 10.3389/fimmu.2022.962986 (PMC9495259; doi:10.3389/fimmu.2022.962986)
Supplement: Supplementary Table 4 — Pathways included for crosstalk analysis. [file Table_4.docx]

| **Table S4** Pathways included for crosstalk analysis | | | |
| --- | --- | --- | --- |
| Pathways | P value | P_BH_ value | DEGs related to FSGS included in the pathways |
| MAPK signaling pathway | 7.30×10^9^ | 1.09×10^7^ | MAX、MAP4K2、MAP3K1、MAP2K3、PPP3CB、MAP3K6、TNFRSF1A、ARRB2、DUSP1、CD14、DAXX、DUSP16、GADD45G、DDIT3、MAPK3、NFKB2、IL1R2、MAPK7、DUSP5、PTPN7 |
| Apoptosis | 3.16×10^6^ | 3.14×10^5^ | PARP4、DAXX、CASP8、CAPN1、CFLAR、GADD45G、BCL2L1、DDIT3、MAPK3、TNFSF10、PARP1、TNFRSF1A |
| Complement and coagulation cascades | 6.64×10^6^ | 6.17×10^5^ | A2M、C3、CLU、F2R、CD59、FGG、F13A1、FGA、serpina1 |
| Endocytosis | 1.85×10^5^ | 1.58×10^4^ | RUFY1、F2R、VPS29、IQSEC1、KIAA0196、ARFGAP3、DNM2、SH3KBP1、ARRB2、AP2A2、ARF1、FAM21C、PLD2、HLA-C、ASAP1 |
| NF-kappa B signaling pathway | 2.22×10^5^ | 1.84×10^4^ | CD14、CD40、CFLAR、PLCG1、BCL2L1、CSNK2B、NFKB2、PARP1、TNFRSF1A |
| Fc gamma R-mediated phagocytosis | 2.22×10^5^ | 1.84×10^4^ | PLCG1、GSN、PLD2、MAPK3、DNM2、FCGR2A、ASAP1、FCGR3A、PRKCD |
| Toll-like receptor signaling pathway | 5.74×10^5^ | 4.28×10^4^ | CD14、IRF3、CD40、CASP8、MAP2K3、IFNAR2、MAPK3、IRF5、IRF7 |
| Natural killer cell mediated cytotoxicity | 6.62×10^5^ | 4.87×10^4^ | PLCG1、IFNAR2、MAPK3、HLA-C、FCGR3B、NFATC2、CD244、TNFSF10、PPP3CB、FCGR3A |
| Protein processing in endoplasmic reticulum | 7.46×10^5^ | 5.41×10^4^ | UGGT1、ERP29、UBE2D3、DERL1、DDIT3、MAN1B1、RPN2、ATF6B、SVIP、PREB、CAPN1 |
| Metabolic pathways | 2.23×10^4^ | 1.45×10^3^ | BCKDHA、GAA、MGAT1、COX6C、PLCB3、UROD、PLCG1、MAT2B、PLD2、ACSS1、NFS1、POLD4、DCTD、ACO1、ACSL6、GLB1、DUT、NTPCR、RPN2、H6PD、GPAT4、SUCLG1、GALK1、LDHA、CECR1、SYNJ1、MDH2、POLE3、FUK、HMGCR、NME2、NME1、MAN1B1、NADK、PTDSS2、PIGT、NOS2 |
| Hematopoietic cell lineage | 5.49×10^4^ | 3.16×10^3^ | CD59、IL7R、HLA-DRB3、CD14、IL1R2、CD3D、CD4 |
| GnRH signaling pathway | 6.61×10^4^ | 3.68×10^3^ | MAP3K1、MAPK3、PRKCD、MAPK7、PLCB3、MAP2K3、PLD2 |
| Phagosome | 8.16×10^4^ | 4.42×10^3^ | FCGR2A、CLEC7A、FCGR3B、FCGR3A、C3、HLA-C、HLA-DRB3、CD14、MARCO |
| Cytokine-cytokine receptor interaction | 9.86×10^4^ | 5.17×10^3^ | CD40、TNFRSF21、IFNAR2、IL7R、IL17RA、PF4、TNFSF10、TNFSF12、CCR6、IL1R2、IL23A、TNFRSF1A |
| T cell receptor signaling pathway | 1.45×10^3^ | 7.16×10^3^ | MAPK3、CD28、PPP3CB、PLCG1、NFATC2、CD3D、CD4 |
| TNF signaling pathway | 1.86×10^3^ | 8.87×10^3^ | MAPK3、CASP8、MAP2K3、ATF6B、CFLAR、TNFRSF1A、RIPK3 |
| Propanoate metabolism | 1.94×10^3^ | 9.14×10^3^ | BCKDHA、LDHA、SUCLG1、ACSS1 |
| Wnt signaling pathway | 1.96×10^3^ | 9.20×10^3^ | LEF1、SIAH1、PLCB3、PPP3CB、WNT10B、CTBP2、CSNK2B、NFATC2 |
| Base excision repair | 2.15×10^3^ | 9.95×10^3^ | POLD4、POLE3、PARP1、PARP4 |
| DNA replication | 2.88×10^3^ | 1.26×10^2^ | POLD4、POLE3、RNASEH1、MCM3 |
| mTOR signaling pathway | 3.02×10^3^ | 1.32×10^2^ | MAPK3、FNIP1、CAB39、TTI1、WNT10B、TNFRSF1A、NPRL3、STRADB |
| Primary immunodeficiency | 3.15×10^3^ | 1.35×10^2^ | CD3D、CD40、CD4、IL7R |
| Allograft rejection | 3.75×10^3^ | 1.56×10^2^ | CD40、HLA-C、CD28、HLA-DRB3 |
| RIG-I-like receptor signaling pathway | 5.21×10^3^ | 2.04×10^2^ | RNF125、MAP3K1、IRF3、IRF7、CASP8 |
| Pyrimidine metabolism | 6.36×10^3^ | 2.38×10^2^ | POLD4、DCTD、NME2、NME1、DUT、POLE3 |
| Phospholipase D signaling pathway | 7.56×10^3^ | 2.72×10^2^ | MAPK3、DNM2、PLCB3、PLCG1、PLD2、ARF1、F2R |
| Cell adhesion molecules (CAMs) | 8.10×10^3^ | 2.88×10^2^ | CD40、HLA-C、CD28、HLA-DRB3、SPN、CD226、CD4 |
| Thyroid hormone signaling pathway | 1.07×10^2^ | 3.59×10^2^ | MAPK3、MED30、MED17、PLCB3、PLCG1、MED12 |
| Neurotrophin signaling pathway | 1.15×10^2^ | 3.77×10^2^ | MAP3K1、MAPK3、PRKCD、MAPK7、PLCG1、ARHGDIA |
| Sphingolipid signaling pathway | 1.19×10^2^ | 3.87×10^2^ | MAPK3、PLCB3、S1PR4、PLD2、TNFRSF1A、S1PR1 |
| Platelet activation | 1.24×10^2^ | 3.99×10^2^ | FCGR2A、MAPK3、PLCB3、FGG、F2R、FGA |
| Citrate cycle (TCA cycle) | 1.28×10^2^ | 4.10×10^2^ | ACO1、SUCLG1、MDH2 |
| Galactose metabolism | 1.38×10^2^ | 4.33×10^2^ | GALK1、GAA、GLB1 |
| VEGF signaling pathway | 1.59×10^2^ | 4.83×10^2^ | PPP3CB、MAPK3、PLCG1 |
