# Supplementary material for: Integrated genomic, transcriptomic and metabolomic analysis reveals MDH2 mutation-induced metabolic disorder in recurrent focal segmental glomerulosclerosis
Source: Front Immunol. 2022 Sep 8;13:962986. doi: 10.3389/fimmu.2022.962986 (PMC9495259; doi:10.3389/fimmu.2022.962986)
Supplement: Supplementary file 5 [file DataSheet_1.pdf]

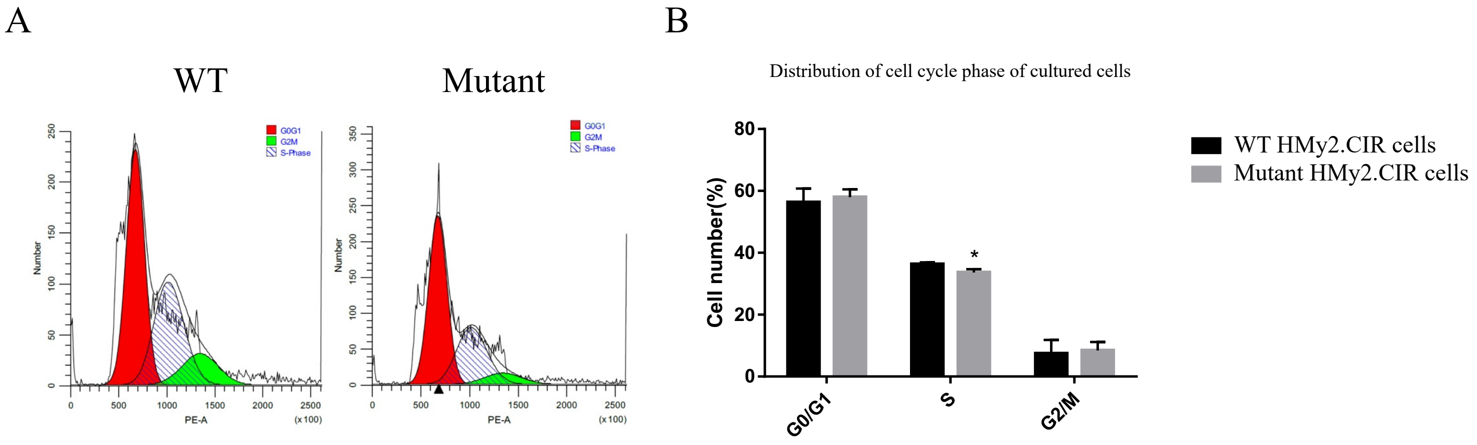

Figure S1. (A) Flow cytometry detectes the distribution of cell cycle phase in WT HMy2.CIR or mutant HMy2.CIR cells ($n = 3$). (B) Statistical results of (A). $^*p < 0.05$; NS, no significance.

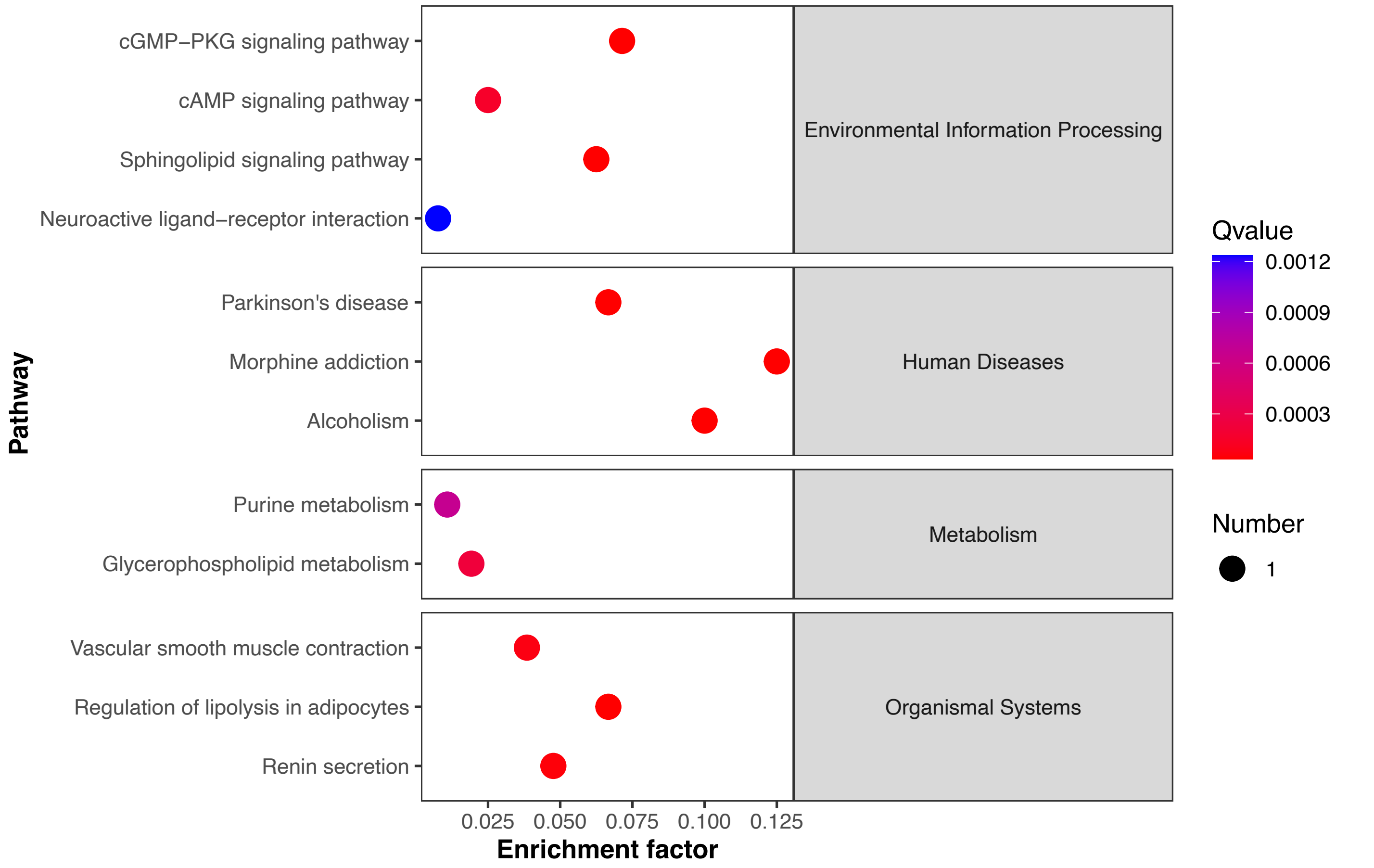

Figure S2. HMy2.CIR cells carrying c.26C>T mutation mutation in MDH2 exhibited metabolic changes. Kegg enrichment results were shown as bubble chart. Enrichment factor indicates the number of differential metabolites located in the KEGG/the total number of metabolites located in the KEGG. The smaller the p value, the higher the enrichment of KEGG.

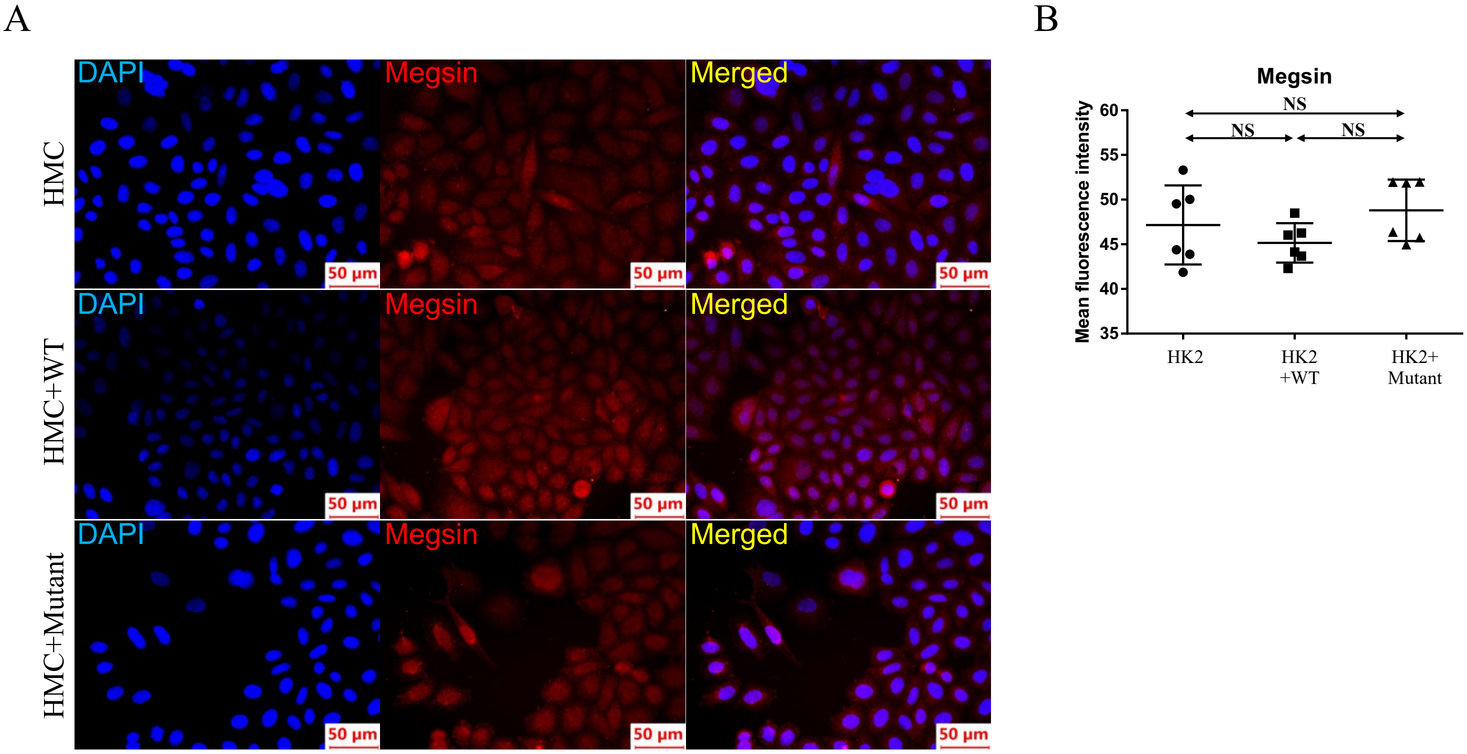

Figure S3. (A-B) Representative pictures and quantification of immunofluorescence staining of megsin (red) and DAPI (blue) in HMC after co-culturing with WT or mutant HMy2.CIR cells. Six random fields were taken from each coverslip (mean ± SD, n = 6). Scale bar = 50 μm. HMC, human mesangial cells; NS, no significance.

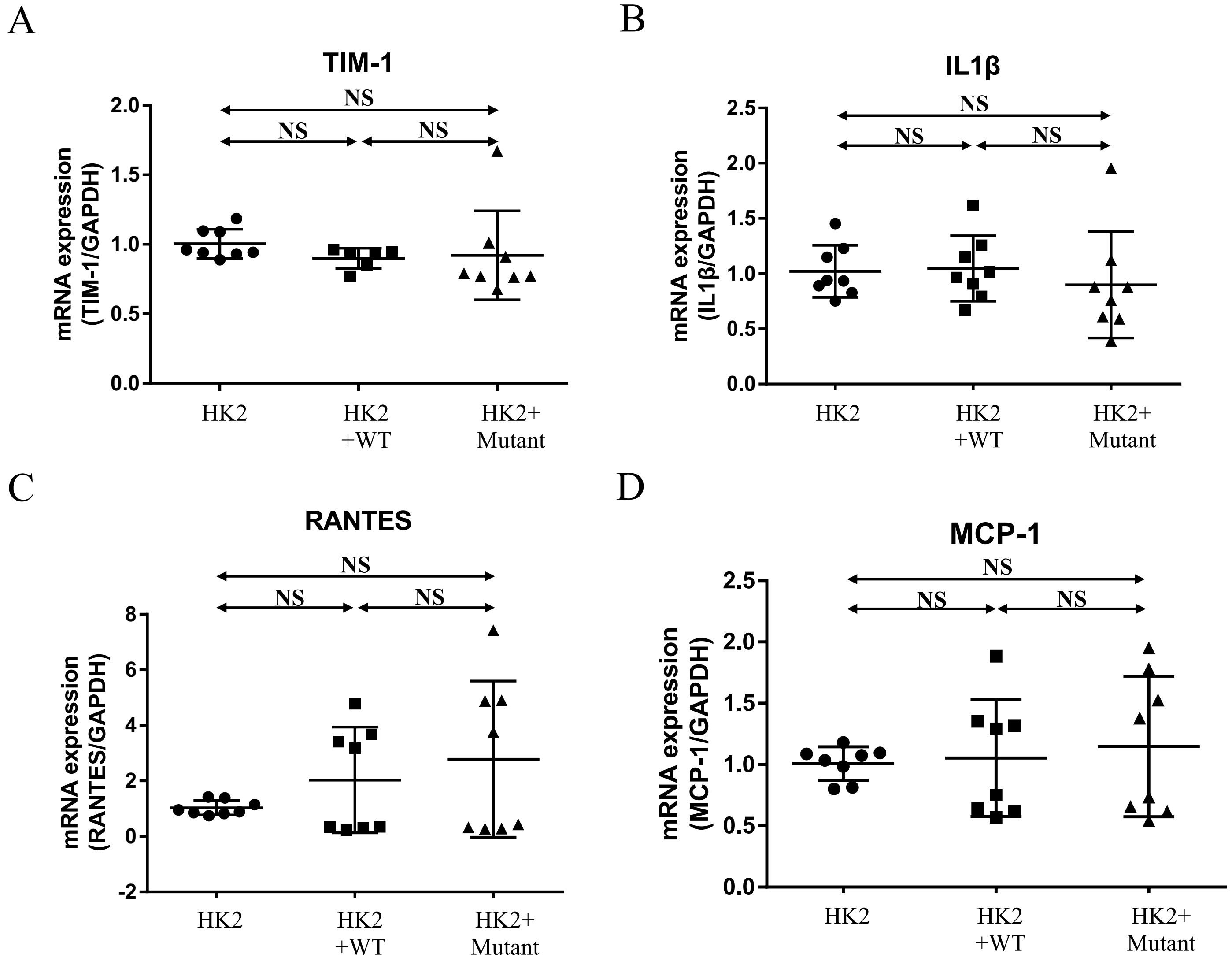

Figure S4. Relative mRNA expression level of (A) TIM-1, (B) IL1β, (C) TANTES and (D) MCP-1 in HK2 cells after co-culturing with WT or mutant HMy2.CIR cells. NS, no significance.
